# The therapeutic threshold in clinical decision-making for TB

**DOI:** 10.1093/inthealth/ihad002

**Published:** 2023-02-06

**Authors:** Madeleine L de Rooij, Lutgarde Lynen, Tom Decroo, Aquiles R Henriquez-Trujillo, Tom Boyles, Bart K M Jacobs

**Affiliations:** Department of Clinical Sciences, Institute of Tropical Medicine, Antwerp, 2000, Belgium; Department of Clinical Sciences, Institute of Tropical Medicine, Antwerp, 2000, Belgium; Department of Clinical Sciences, Institute of Tropical Medicine, Antwerp, 2000, Belgium; Facultad de Medicina (Faculty of Health Science), Universidad de Las Américas, Quito, 170125, Ecuador; Division of Infectious Diseases, Helen Joseph Hospital, Johannesburg, 2092, South Africa; Perinatal HIV Research Unit (PHRU) at the University of the Witwatersrand, Johannesburg, 1864, South Africa; Infectious and Tropical Diseases, London School of Hygiene and Tropical Medicine London, London, WC1E 7HT, UK; Department of Clinical Sciences, Institute of Tropical Medicine, Antwerp, 2000, Belgium

**Keywords:** clinical decision-making, diagnosis, threshold, TB

## Abstract

Because TB control is still hampered by the limitations of diagnostic tools, diagnostic uncertainty is common. The decision to offer treatment is based on clinical decision-making. The therapeutic threshold, test threshold and test-treatment threshold can guide in making these decisions. This review summarizes the literature on methods to estimate the therapeutic threshold that have been applied for TB. Only five studies estimated the threshold for the diagnosis of TB. The therapeutic threshold can be estimated by prescriptive methods, based on calculations, and by descriptive methods, deriving the threshold from observing clinical practice. Test and test-treatment thresholds can be calculated using the therapeutic threshold and the characteristics of an available diagnostic test. Estimates of the therapeutic threshold for pulmonary TB from intuitive descriptive approaches (20%–50%) are higher than theoretical prescriptive calculations (2%–3%). In conclusion, estimates of the therapeutic threshold for pulmonary TB depend on the method used. Other methods exist within the field of decision-making that have yet to be implemented or adapted as tools to estimate the TB therapeutic threshold. Because clinical decision-making is a core element of TB management, it is necessary to find a new, clinician-friendly way to unbiasedly estimate context-specific, agreed upon therapeutic thresholds.

## Introduction

Although there has been an overall reduction in TB cases (Global TB report 2020),^1^ control is still hampered by the limitations of diagnostic tools. The accepted definition of active TB disease is a combination of compatible symptoms with a diagnostic test showing microbiological evidence of *Mycobacterium tuberculosis* (MTB) in a clinical sample, although it is also accepted that patients can be asymptomatic or can be diagnosed without microbiological confirmation, particularly in paucibacillary disease such as tuberculous meningitis. Among confirmatory tests, culture is time consuming and results are often not available when clinical decisions are required, while smear microscopy has low sensitivity. Recently introduced nucleic acid amplification tests are faster and are a reasonable proxy for culture with a sensitivity and specificity of 73%–91% and 96%–98%, respectively, compared with culture.^2,3^

Despite these advances, TB diagnosis is often hampered by a lack of availability of specimens, for example, due to sputum scarcity and because appropriate tests are not available in all settings. As a result, diagnostic uncertainty is common at the time that a decision whether to treat for TB is required.

A clinical decision-making process based on the level of diagnostic certainty and benefits and harms of each decision is required.^4^ Clinicians typically estimate the probability that a patient has the disease and weigh the harm and benefit of treating and not treating using intuition and experience. It can be made more explicit by applying the threshold approach, as introduced by Pauker and Kassirer.^5^ The key concept is the therapeutic threshold, which is applicable when a clinician is faced with a decision to treat or not treat for a condition when no further testing is available. They developed a method based on expected utility theory (EUT), aiming to choose the option with the highest expected utility, calculated as the sum of utilities of all possible outcomes of an action weighted by their corresponding probabilities.^6,7^

EUT takes into account the net utility (or ‘value’) of treating when the disease is present (${U}_{T|D}$) or absent (${U}_{T|no\ D}$), and not treating when the disease is present (${U}_{no\ T|D}$) or absent (${U}_{no\ T|no\ D}$). Table S3 shows the calculation.

The therapeutic threshold is the probability of disease (p_D_) at which the expected utility of treating (EU_T_) and not-treating (EU_no T_) is the same, resulting in the clinician having no preference on which decision to take.

**Figure 1.**
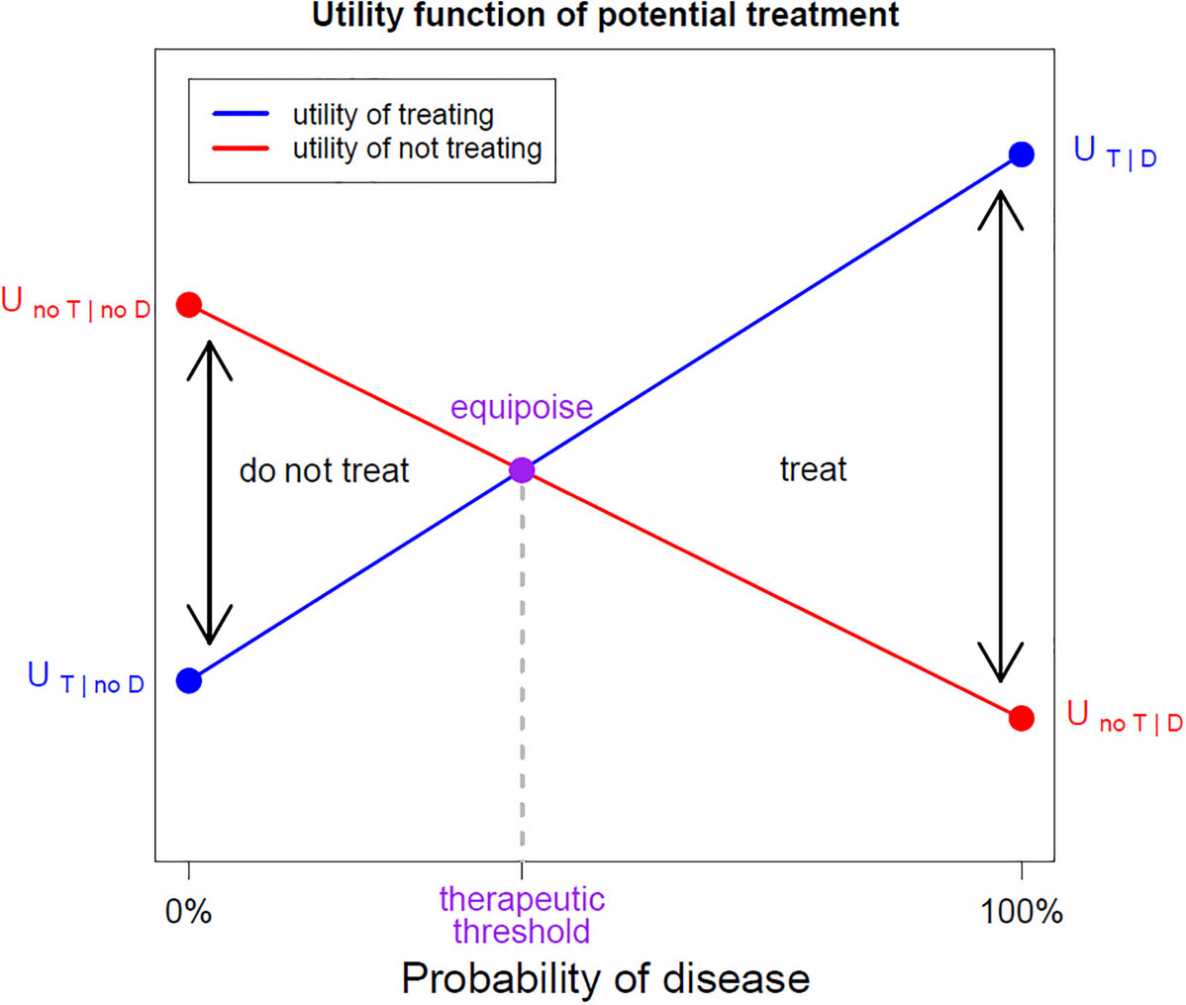
Utility function of potential treatment. The expected utility of treat and no treatment options are shown as a function of the probability of disease, inspired by Sox et al.^4^ A patient either has or does not have the disease, and is treated or not treated. The four combinations of true status and decision taken are shown as dots in the graph. When the true disease status is not known, it is possible to estimate the probability that a patient has the disease and estimate the expected utility of, respectively, treating (blue line) and not treating (red line). The therapeutic threshold is the probability of disease at which the expected utilities are equal. The expected utility theory (EUT), aimed at choosing the option with the highest expected utility, is calculated as the sum of utilities of all possible outcomes of an action weighted by their corresponding probabilities. EUT takes into account the net utility (or ‘value’) of treating when the disease is present (U_(T|D)_) or absent (U_(T|no D)_), and not treating when the disease is present (U_(no T|D)_) or absent (U_(no T|no D)_). The therapeutic threshold is the probability of disease at which the expected utility of treating and not treating is the same.

**Figure 2.**
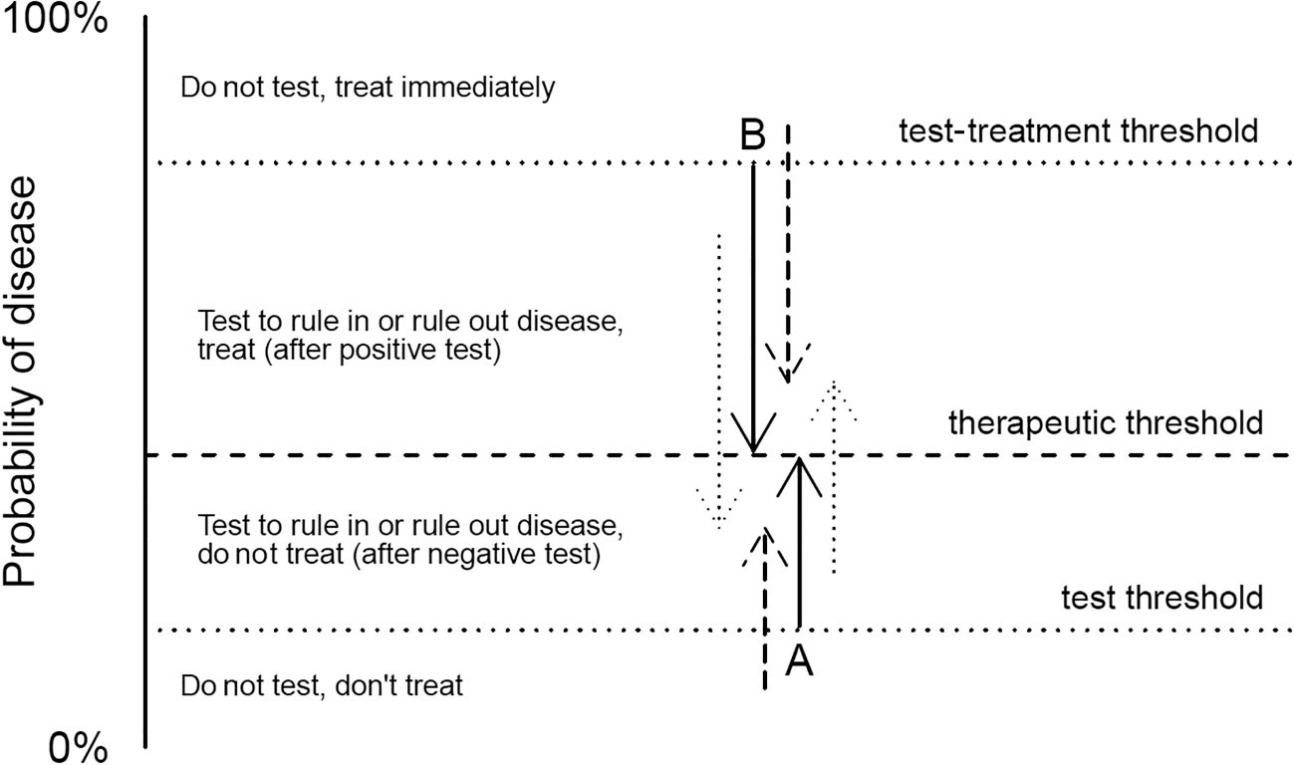
Test threshold and test-treatment threshold. The test and test-treatment thresholds and the need for a diagnostic test is shown, as inspired by Decroo et al.^10^ A probability of disease equal to the test threshold before testing will result in a probability equal to the therapeutic threshold if the test is positive (arrow A). Similarly, a probability of disease equal to the test-treatment threshold before testing will result in a probability equal to the therapeutic threshold if the test is negative (arrow B). Consequently, when the probability of disease is below the test threshold or above the test-treatment threshold, the test result does not bring sufficient evidence to impact the treatment decision (dashed arrows). Only when the probability of disease lies between the two thresholds should the test be performed and its result followed (dotted arrows).

Figure 1 shows the concepts of the therapeutic threshold, Figure 2 the test threshold and test-treatment threshold. In Figure 1, the expected utility of treat and no treatment options are shown as a function of the probability of disease (pD), inspired by Sox et al.,^4^ as the equations below.


\begin{eqnarray*} E{U}_T &=& \ {p}_D{U}_{T|D} + \left( {1 - {p}_D} \right){U}_{T|no\ D}\nonumber\\ E{U}_{no\ T} &=& \ {p}_D{U}_{no\ T|D} + \left( {1 - {p}_D} \right){U}_{no\ T|no\ D}\nonumber\\ E{U}_T &=& E{U}_{no\ T}\ \Leftrightarrow \ {p}_D = \frac{{\left( {{U}_{no\ T|no\ D} - {U}_{T|no\ D}} \right)}}{{\left( {{U}_{no\ T|no\ D} - {U}_{T|no\ D}} \right) + \left( {{U}_{T|D} - {U}_{no\ T|D}} \right)}}\end{eqnarray*}

Only when the probability of disease is higher than the therapeutic threshold, then the expected utility of treatment is higher than of non-treatment and treatment should be started.^5^ Therapeutic thresholds are lower when patients are in a worse clinical condition, because the consequences of withholding treatment are greater than when a patient is less ill.^8^

Pauker and Kassirer introduced two further thresholds based on the availability of a final diagnostic test with imperfect accuracy, a situation that is common in TB, namely, a test-treatment threshold and a testing threshold.^9^ The test threshold is the probability of disease where the clinician is at equipoise regarding the decision to rule out the disease or collect extra information by performing a test, and the test-treatment threshold is the probability of disease where there is no preference between gathering additional information by a test and to rule in disease and start treatment.

In fact, this approach divides the former therapeutic threshold into two separate thresholds. The test and the test-treatment thresholds can be derived from the therapeutic threshold and the diagnostic accuracy of a single test, using either Bayes’ theorem^10–12^ or a direct derivation based on utilities. Compared with the therapeutic threshold, the utilities are now weighed by properties of the test, respectively, the positive likelihood ratio and the negative likelihood ratio. This is shown in the equations below and Figure 1.


\begin{eqnarray*}
E{U}_{Test} &=& Se\ {p}_D{U}_{T|D} + \left( {1 - Sp} \right)\left( {1 - {p}_D} \right){U}_{T|no\ D}\nonumber\\&& + \ \left( {1 - Se} \right){p}_D{U}_{no\ T|D} + Sp\left( {1 - {p}_D} \right){U}_{no\ T|no\ D}\end{eqnarray*}

\begin{eqnarray*}
E{U}_{no\ T} &=& E{U}_{Test}\ \mathop \Leftrightarrow \limits_{} \ \nonumber\\
{p}_D &=& \frac{{\left( {1 - Sp} \right)\ \left( {{U}_{no\ T|no\ D} - {U}_{T|no\ D}} \right)}}{{\left( {1 - Sp} \right)\left( {{U}_{no\ T|no\ D} - {U}_{T|no\ D}} \right) + Se\left( {{U}_{T|D} - {U}_{no\ T|D}} \right)}}\ \end{eqnarray*}

\begin{eqnarray*}
E{U}_T &=& E{U}_{Test}\ \mathop \Leftrightarrow \limits_{} \ \nonumber\\
{p}_D &=& \frac{{Sp\left( {{U}_{no\ T|no\ D} - {U}_{T|no\ D}} \right)}}{{Sp\left( {{U}_{no\ T|no\ D} - {U}_{T|no\ D}} \right) + \left( {1 - Se} \right)\ \left( {{U}_{T|D} - {U}_{no\ T|D}} \right)}}\ \end{eqnarray*}

Following this equation, the probability of disease before performing a test and the diagnostic accuracy of the test together determine if it is useful to perform a test.

As illustrated in Figure 2, only when the probability of disease lies between the two thresholds should the test be performed and its result followed. However, when the test itself has a non-negligible cost or may be a direct cause of harm, its use may still be inferior, despite the probability lying between these boundaries. This exception is relevant for invasive tests or in settings with limited testing resources, and will typically apply to presumptive patients with a probability of disease that only barely exceeds the test threshold (the ‘wait and see’ approach).

The therapeutic, test and test-treatment thresholds are key in guiding clinical decision-making in TB. This also makes it possible to predefine the targets of diagnostic accuracy and the pretest probability needed in order for a test to make a difference.^13^ Hence, determining the therapeutic threshold is essential to make decisions about diagnostic tests.

The formulas above are generic, with the utilities needing to be estimated. Two examples are shown in the supplementary information (Supplementary Data 1, with Tables S1-S4). Next to a simple intuitive guess, several methods have been used to estimate the optimal or actual therapeutic threshold. There is no consensus on the best approach. Prescriptive methods, such as EUT, are based on calculations where the utilities in the formula are quantified based on mortality and morbidity. Descriptive methods meanwhile derive the threshold estimate from observing clinical practice under the assumption that program managers and physicians intuitively weigh the utilities when developing guidelines and taking treatment decisions.

While there has been extensive research on methods to estimate action thresholds, only one review on the use of the therapeutic threshold in TB exists to the best of our knowledge, and it was limited to prescriptive methods. In this review we briefly summarize some of the literature on different methods used to estimate the therapeutic threshold in general, what has been done for pulmonary TB specifically and identify the existing research gaps.

## Methods

We searched for studies of methods for estimating the therapeutic threshold using bibliographic and snowball (or citation) search methods. Our initial search was unrestricted by condition and a subsequent search specified TB. For the latter we used the following search string on PubMed: ‘((therapeutic and threshold) or ‘test threshold’) and (TB or tuberculosis)’

There are no existing MeSH terms for ‘therapeutic threshold’, nor other terms used for the same principle such as ‘treatment threshold’ and ‘threshold approach’. We therefore used the all fields-search string ‘threshold AND (therapeutic OR treatment)’ in PubMed for our initial search and ‘threshold AND (therapeutic OR treatment) AND tuberculosis [Mesh]’ for the TB-specific search.

After reading the title and/or abstract, articles were retrieved if they showed data on methods for the calculation of thresholds for clinical decision-making. After reading the full text, articles were excluded if they only addressed clinical decision-making for pediatric patients.

## Results

**Figure 3.**
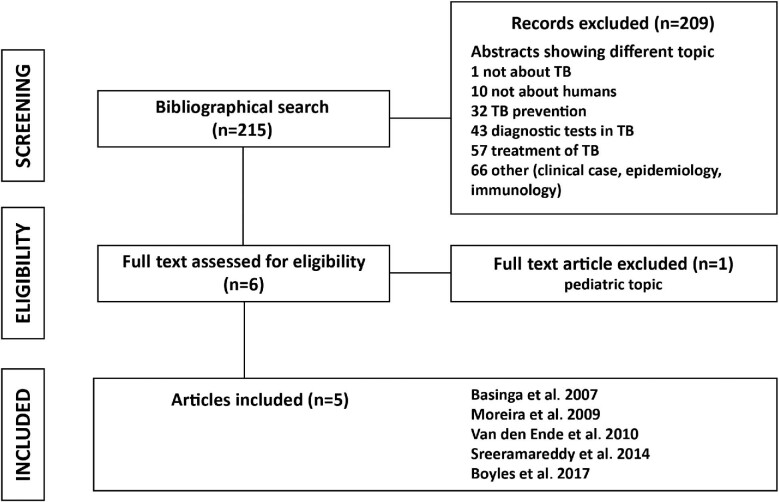
Flowchart showing articles retrieved at different steps of the literature review. n, number.

The PubMed literature search for articles concerning methods to estimate the therapeutic threshold (not limited to TB) yielded >100 000 individual citations. The PubMed literature search was further refined for studies addressing the therapeutic threshold in TB and this yielded 215 records; 210 were considered not relevant after reviewing the title and abstract. The snowball search found the same five articles. The search for articles about the therapeutic threshold in TB is summarized in Figure 3. The characteristics of the five articles are summarized in Table 1.

**Table 1.** Methods used by retrieved studies of the therapeutic threshold for TB

First author, year of publication	Country of participants	Method used to estimate the TB therapeutic thresholds
Basinga, 2007	Rwanda	• intuitive threshold • calculated weights based on intuitive probabilities • calculated weight based on literature probabilities
Moreira, 2009	Ecuador, Laos, Nepal and Rwanda	• intuitive threshold • calculated weights based on intuitive probabilities • calculated weight based on literature probabilities
Van den Ende, 2010	Participants of courses (names of countries not specified)	• intuitive threshold • calculated weights based on intuitive probabilities
Sreeramareddy, 2014	India, Pakistan and Bangladesh	• intuitive threshold • calculated weights based on intuitive probabilities • calculated weight based on literature probabilities
Boyles, 2017	South Africa	• Clinical vignettes to calculate thresholds

Using prescriptive methods, Basinga et al.^14^ and Sreeramareddy et al.^15^ estimated the therapeutic threshold in TB in Rwanda, and India, Pakistan and Bangladesh, respectively (Table 2). Healthcare professionals were asked to estimate an intuitive threshold and intuitively choose factors playing a role in the location of the threshold. Both studies show a clear difference between the intuitive threshold (52.5% and 25%, respectively) and the calculated thresholds based on EUT (2.7%–11.9% and 2.0%–5.9%, respectively).

**Table 2.** Estimates of therapeutic thresholds for TB

Therapeutic threshold	Basinga et al.^14^ (n=28)	Sreeramareddy et al.^15^ (n=242)
Intuitive	52.5%	25.0% (16.7–41.7)
Calculated with intuitive determinant factors (including cost and regret)	11.9%	2.0% (0.8–7.5)
Calculated with intuitive determinant factors (without cost and regret; restrictive threshold)	2.8%	2.9% (1.0–8.3)
Computed with determinant factors derived from literature (without cost and regret; restrictive threshold)	2.7%	5.9% (3.7–7.7)

Abbreviation: n, number.

These studies, as well as an article by Van den Ende et al. based on the intuitive determinant factors of Basinga et al.’s study,^16^ show that the published therapeutic threshold for smear-negative TB based on EUT hovers around 2%–3%, while most physicians say they would not treat a patient when the probability is lower than 20%–50%.

One other study performed by Moreira et al.^17^ in Ecuador, Nepal, Laos and Rwanda also asked clinicians to estimate the probability of harmful outcomes of TB and TB treatment, while Sreeramareddy et al. and Moreira et al. also asked participants to weigh harmful outcomes of TB disease and TB treatment (Tables S1 and S2). It is still unclear if physicians weigh provoked harm from treatment higher than harm due to the disease, which would require an adaptation of the classical EUT model.^18^

If the therapeutic threshold is impacted by unquantifiable cognitive and emotional factors, it can only be estimated unbiasedly by descriptive methods instead.

Recently, Boyles et al. has estimated therapeutic thresholds for TB in patients infected with HIV using web-based clinical vignettes.^8^ A series of hypothetical scenarios with randomly varied probabilities of TB was presented to participants, who stated their decision to test, treat or do neither for each case. The therapeutic threshold was estimated from the decisions taken by participants for the different scenarios, being followed by the test and test-treatment thresholds calculated as explained before and using the characteristics of Xpert MTB/RIF. Critical factors defining the therapeutic threshold were the severity of disease (with possible TB-meningitis having the lowest threshold) and the clinical stability of the patient. The thresholds were lower for more severely ill patients, when implications of a false negative diagnosis are more serious. The therapeutic threshold was near 0% for an unstable patient with possible TB-meningitis and 3.4% for an unstable inpatient with possible pulmonary TB or extrapulmonary TB outside the brain, but between 70% and 80% for a stable patient (Table 3).

**Table 3.** Therapeutic threshold, test threshold and test-treatment threshold for TB in patients with HIV, using Xpert MTB/RIF as diagnostic test

	Therapeutic threshold	Test threshold	Test-treatment threshold
Inpatient, possible PTB or EPTB >Stable	79.6%	8.2%	97%
>Unstable	3.4%	0.08%	23%
Outpatient with possible PTB >Stable	74.5%	6.2%	96%
>Unstable	29.1%	0.92%	77%
Possible TB-meningitis >Stable	51.4%	2.2%	0.7%
>Unstable	0%	0%	0%

Abbreviations: EPTB, extrapulmonary TB; PTB: pulmonary TB.

## Discussion

To make appropriate clinical decisions, it is important to estimate the therapeutic threshold, the probability of disease at which the expected value of treating and not treating are the same. In the field of TB, where diagnostic uncertainty is common, this could prevent overtreatment and undertreatment, improving morbidity and mortality and reducing costs.

The therapeutic threshold in TB was estimated with prescriptive methods, based on calculations and by descriptive methods, deriving the threshold from observing or mimicking clinical practice. The test and test-treatment thresholds were estimated using the estimate of the therapeutic threshold and the characteristics of the routinely available diagnostic test in South Africa.

The mentioned studies about prescriptive methods to estimate the therapeutic threshold in TB show a clear difference between intuitive estimates and the therapeutic threshold calculated with EUT. The intuitive threshold is between 20% and 50%, while the therapeutic threshold for smear negative TB based on EUT hovers around 2%–3%.^14–16^ The two estimated restrictive thresholds (one using the intuitive determinant factors and the other based on probabilities derived from the literature) are similar, suggesting that there is no substantial difference between clinicians’ estimates of the probabilities of influencing factors and the literature, but that the difference between EUT-derived and intuitive thresholds occurs when the factors are integrated towards a threshold.^14,15^

There are several possible explanations for this difference between intuitive and prescriptive thresholds. A difference in estimation of the probability of outcomes of TB disease and treatment leads to different thresholds. In the case of a wide range of estimated probabilities between different groups, this can explain the difference in therapeutic thresholds between the groups. For example, a higher estimated probability of harmful treatment outcomes (as in the studies of Basinga et al., Sreeramareddy et al. and Moreira et al.^14,15,17^) increases the therapeutic threshold. Because it is difficult to estimate the probability of rare events, such as treatment mortality, it might be easier to estimate mortality on an individual basis by using prediction rules based on, for example, danger signs.^19^

A second possible explanation for differences between calculated and intuitive therapeutic thresholds is a different weight given to a harmful outcome of disease and treatment. For a serious (and often lethal) condition, such as active TB, the harm of omission (allowing harm from the disease by not treating a diseased person) is higher than the harm of commission (actively inflicting harm, e.g. by prescribing an unjustified treatment). However, in TB, clinicians seem to give the harm of a provoked unjustified death a higher weight than the harm of a provoked justified death,^15,17^ meaning that, independent of objective factors, they consider it more harmful to commit than to omit, hence increasing the intuitive therapeutic threshold. This is related to the regret factor,^20^ also demonstrated by Basinga et al., showing a clear difference in the threshold calculated with and without taking regret into account (11.9% and 2.8%, respectively).^14^ The acceptable regret approach to clinical decision-making takes this into account.^21^ When this approach is applied to TB, the anticipated number of wrongly treated patients would be too high to be acceptable for physicians.^22^ However, physicians are very different in the way they deal with harm, being either more or less risk averse.^22^ Because EUT has difficulties with incorporating these different values regarding outcomes related to harm and benefit, this is a possible explanation for the difference between EUT-based and intuitive thresholds.

Furthermore, the intuitive therapeutic threshold is influenced by other cognitive and emotional factors; for example, the high pressure on healthcare facilities and resources is, in particular, considered an important factor in low-resource settings. One can argue that EUT overlooks these factors, as they are unmeasured and no formula can take the human multidimensional reflective rationality fully into account.^22^

It is important to realize that thresholds are context-specific and to carefully select the participants of interest. The societal context, available resources, cultural factors and individual morals with respect to judgment of possible outcomes of the action (among them regret) all influence the expected values of treating and non-treating.^12,17,21^ Knowledge, work experience, level of education and working field (clinical or public health)^14^ may play a role as well.

Finally, differential diagnosis may also play a role. Because most symptoms are not specific for TB, someone with a probability of TB of 5% may show a clinical presentation that is more in line with an alternative diagnosis and associated treatment that should be pursued first. While EUT can and should be adapted to take the harm of missing a differential diagnosis into account, this is not trivial.

It is therefore claimed as fact that physicians do not act according to EUT.^23^ We believe this is not necessarily true. Rather, EUT is likely not applied correctly when only morbidity and mortality are taken into account. The impact of overtreatment corresponding to EUT-derived thresholds would have numerous negative consequences on the healthcare system in resource-challenged settings. If the additional, hard-to-quantify harms of overtreatment and associated loss of utility of treating are included, EUT may actually match intuitive thresholds.

Compared with the prescriptive methods, descriptive methods are not directly based on estimations of probabilities and weight of outcomes, but instead try to model actual treatment decisions, which implicitly take cognitive and emotional factors into account. Hence, descriptive methods may better reflect real-life clinical decisions. Feedback by colleagues may not be possible although in daily life clinicians often use discussions with and the feedback of colleagues to reach a decision. Additionally, a correct estimation of the probability of TB for suspect cases is not trivial. Furthermore, a disadvantage of descriptive methods is that it is not possible to analyze the steps clinicians have taken in their decision-making, because the only result is the final treatment decision.

Finally, the actual threshold is unknown, and it is unclear how closely the answers given to such vignettes match the true clinical reality. Physicians are unlikely to be immune to social desirability bias and other cognitive biases when responding to surveys. Therefore, because these methods aim to describe clinical practice, it is unclear if the resulting threshold is an estimate of the desired threshold or rather of the existing threshold following directly from potentially suboptimal guidelines that are currently in place.

A variety of other methods exist to aid or optimize decision-making in a variety of fields. To the best of our knowledge, these methods have not yet been adapted to estimate the therapeutic threshold in TB. It is not inconceivable that applications to estimate other action thresholds can be readily adapted for this purpose.

When outcomes are described as quality-adjusted life-years, any extension of the Von Neumann-Morgenstern utility theorem can be considered.^24^ In addition to basic EUT, the standard gamble and time trade-off techniques have been studied extensively in the field of health economics.^25–27^ Adaptations to estimate the therapeutic threshold with these techniques are potentially straightforward but would be expected to retain some of the limitations of classic EUT.

Methods from the field of multiple criteria decision analysis can probably be adapted to estimate treatment preferences as well.^28^ Both swing weighting and discrete choice experiments have been studied for this purpose in the context of drug development.^29^

Formal consensus development methods can be used to propose an intuitive therapeutic threshold. These methods capture collective knowledge by guiding a group discussion following a standardized procedure and translate this into a collective (medical) decision.

Formal consensus development methods have been used in the health sector since the 1950s,^30,31^ mainly for the development of guidelines and exploration of moral and ethical questions. The nominal group technique, Delphi method and consensus development conference are the most popular methods.

These methods generally use an iterated process providing feedback of group opinion after each round. The group dynamic leads to opinion-building and decision-making.^32,33^

The use of anonymity limits social and psychological pressure, reduces the effect of dominant individuals and usually increases the response rate.^32^

To use the therapeutic threshold in decision-making in the framework of TB guideline development (whether at the national or hospital level), it is necessary to find an easier and more accurate way to estimate a context-specific threshold than that provided by currently existing methods. Several methods from other fields currently remain unexplored for clinical decision-making in general and TB in particular.

In conclusion, after a literature search, we found five papers that estimated the therapeutic threshold in TB. We have described how different methods were used to estimate thresholds used for clinical decision-making in the field of TB. Prescriptive methods estimate the threshold using formulas based on the weight of influencing factors. Descriptive methods may represent the real-life situation more accurately, but can be biased in the direction of existing guidelines. Other methods exist but have not been applied and may be promising alternatives to the existing approaches. The currently non-existing ideal method would give accurate and reliable estimates of the therapeutic threshold that can be agreed upon by different stakeholders, and could be efficiently performed in different settings as a first step before diagnostic guideline development.

## Supplementary Material

ihad002_Supplemental_File

## Data Availability

Not applicable for this review.
