# Supplementary material for: The therapeutic threshold in clinical decision-making for TB
Source: Int Health. 2023 Feb 6;15(6):615–22. doi: 10.1093/inthealth/ihad002 (PMC10629962; doi:10.1093/inthealth/ihad002)
Supplement: ihad002_Supplemental_File [file ihad002_supplemental_file.docx]

The therapeutic threshold in clinical decision making for TB
Supplementary information

**AUTHORS:** Madeleine L. de Rooij^1^, Lutgarde Lynen^1^, Tom Decroo^1,2^, Aquiles R. Henriquez-Trujillo^3^, Tom Boyles^456^, Bart K.M. Jacobs^1*^

1. Institute of Tropical Medicine, Antwerp, Belgium
2. Research Foundation Flanders, Brussels, Belgium
3. Universidad de Las Américas, Quito, Ecuador
4. Division of Infectious Diseases, Helen Joseph Hospital, Johannesburg, South Africa
5. Perinatal HIV Research Unit (PRHU) at the University of the Witwatersrand, Johannesburg, South Africa
6. London School of Hygiene and Tropical Medicine London, United Kingdom

## **Weighing harmful outcomes**

Multiple studies estimated the weight of harmful outcomes, summarized in the tables below.

Table S1 Estimations of interviewed clinicians of probability of outcomes of disease and treatment and probabilities derived from literature (all median with IQR)

|  | **Basinga**(*15*) | **Sreeramareddy**(*16*) | **Moreira**(*17*) | **Literature** |
| --- | --- | --- | --- | --- |
| Disease mortality | 75% (50-85) | 65% (50-75) | 60% (50-85) | 55% |
| Disease morbidity | 22.5% (15-25) | 20% (8-30) | 20% (10-43) | 19% |
| Treatment mortality | 1% (0.28-1) | 0.7% | 0.5% (0.23-2) | 0.09% |
| Treatment morbidity | 5% (2-11.25) | 1.5% | 1% (0.4-3) | 4.9-5.8% |

Table S2 Weight of harmful outcomes of TB-disease and -treatment (morbidity: the complement of the health status of a person affected by the disease compared with a healthy person of the same age (in % with IQRs), death: scaled 0-10)

|  | **Moreira**(*17*) | **Sreeramareddy**(*16*) |
| --- | --- | --- |
| Disease morbidity | 70 (50-90) | 25 (15-40) |
| Treatment morbidity | 70 (42.5-90) | 60 (40-80) |
| Death due to omission | 5 (3-6.5) | 4 (3-5) |
| Provoked unjustified death | 3 (2-5) | 3 (2-4) |
| Provoked justified death | 1 (1-2) | 1 (1-2) |

## **Therapeutic threshold formula**

Table S3 Calculations of the therapeutic threshold

| Calculated threshold The generic formula for the therapeutic threshold is equal to:  $p_{D}=\frac{(U_{no T\vert no D}-U_{T\vert no D})}{\left( U_{no T\vert no D}-U_{T\vert no D} \right)+(U_{T\vert D}-U_{no T\vert D})}$  While the utilities could theoretically be estimated separately, it is usually easier to consider the differences.  The first difference is $U_{T\vert D}-U_{no T\vert D}$, the difference in utility between treating and not treating when a patient has true TB. This is therefore the net benefit of treatment for a true TB case, and could be quantified as the avoided mortality and morbidity by treating, or alternatively the expected quality adjusted life years (QALYs) gained from treating.  The second difference is $U_{no T\vert no D}-U_{T\vert no D}$, the difference in utility between not treating and treating a patient that does not have TB. This is the foregone harm by not exposing a person to treatment who does not need it, and could be quantified as the avoided mortality and morbidity from not exposing a person unnecessarily to treatment, or alternatively the expected QALYs lost due to toxicity and side-effects.  For example, in Basinga et al, using estimates derived from the literature (Table S1), the first difference is calculated as the sum of the avoided mortality (median 55%), the avoided morbidity (median 14% = 19%-5%) which is weighed as 25% as severe as mortality, and the reverse of the mortality from treatment failure (10%). This would be give a total treatment benefit of 48.5% (55%+14%*0.25-10%) for people with true TB.  The second difference is simply the mortality (0.1%) and morbidity (4.9%) of treatment, for a total avoided harm of 1.3% (0.1%+4.9%*0.25) if no treatment is unnecessarily given.  The therapeutic threshold would then be the latter number divided by the sum of both:  $\frac{1.3\%}{48.5\%+1.3\%}= 2.6\%$ Intuitive weighing We recently did an informal exercise with a few health care professionals with experience in TB, including two of the authors of this manuscript, that included weighing the harms of treatment mortality and morbidity for a true negative TB case versus those of not treating a true positive TB case through consensus building. The resulting ratio was about 1 to 3. Plugging this in the formula as avoided harms would result in a threshold of 25% (1/(3+1)).  Of note, the much higher estimate is linked to the consideration that people who are wrongly put on treatment usually stay on treatment, while those who are not given treatment could often be followed up and given treatment at a later stage when more evidence presents itself, or differential diagnoses have been ruled out.  When other, harder to quantify factors like transmission, stigma, cost and loss of trust were give an intuitive weight as well, the ratio was approximately 1 to 2 for a threshold of 36%. |
| --- |

## **Comparison between consensus development methods**

Table S3 Characteristics of formal consensus development methods (adapted from Murphy et al.) (32)

|  | **Delphi** | **NGT** |
| --- | --- | --- |
| Mailed questionnaires | Y | N |
| Private decisions elicited | Y | Y |
| Formal feedback of group judgments | Y | Y |
| Face-to-face contact | N | Y |
| Structured interaction | Y | Y |
| Statistical aggregation method | Explicit | Explicit |
